# Supplementary material for: The Expression of miRNAs in Human Ovaries, Oocytes, Extracellular Vesicles, and Early Embryos: A Systematic Review
Source: Cells. 2019 Dec 4;8(12):1564. doi: 10.3390/cells8121564 (PMC6952888; doi:10.3390/cells8121564)
Supplement: Supplementary file 1 [file cells-08-01564-s001.zip › Appendix S1_vdef.docx]

**Appendix S1.** Search strategy for the literature published between the earliest available online indexing year and June 2018 in the MEDLINE-Pubmed database and EMBASE.

1. MEDLINE-Pubmed
   1. Search terms:

(((((((((((((((((((((((((((((((((((((((((("fertility") OR "infertility") OR "male fertility") OR "male infertility") OR "female fertility") OR "female infertility") OR "sperm dysfunction") OR "sperm dysfunctions") OR "sperm dna damage") OR "varicocele") OR "obstructive azoospermia") OR "non obstructive azoospermia") OR "noa") OR "oat") OR "asthenozoospermia") OR "oligozoospermia") OR "oligoasthenozoospermia") OR "oligoasthenoteratozoospermia") OR "teratozoospermia") OR "live birth") OR "ongoing pregnancy") OR "clinical pregnancy") OR "miscarriage") OR "multiple pregnancy") OR "recurrent miscarriage") OR "implantation rate") OR "cryopreservation rate") OR "aborted embryo") OR "aborted fetus") OR "embryo quality") OR "fertilization rate") OR "fertilization in vitro") OR "in vitro fertilization") OR "intracytoplasmic sperm injection") OR "assisted reproduction technologies") AND "mirna") OR "microrna") OR "ncrna") OR "micro ribonucleic acid") OR "pre mirna") OR "non coding rna") OR "small rna") OR "small noncoding rna"

- 1. Inclusion filters:

Case Reports, Classical Article, Clinical Conference, Clinical Study, Clinical Trial, Clinical Trial, Phase I, Clinical Trial, Phase II, Clinical Trial, Phase III, Clinical Trial, Phase IV, Comparative Study, Controlled Clinical Trial, Dataset, English Abstract, Evaluation Studies, Introductory Journal Article, Journal Article, Meta-Analysis, Multicenter Study, Observational Study, Pragmatic Clinical Trial, Randomized Controlled Trial, Validation Studies, Abstract, Humans, English, Male and Female.

1. EMBASE
   1. Search terms:

('fertility':ab,ti OR 'infertility':ab,ti OR 'male fertility':ab,ti OR 'male infertility':ab,ti OR 'female fertilty':ab,ti OR 'female infertility':ab,ti OR 'sperm dysfunction':ab,ti OR 'sperm quality':ab,ti OR 'sperm dna damage':ab,ti OR 'non obstructive azoospermia':ab,ti OR 'asthenospermia':ab,ti OR 'oligospermia':ab,ti OR 'oligoasthenozoospermia':ab,ti OR 'oligoasthenoteratozoospermia':ab,ti OR 'spermatozoon abnormality':ab,ti OR 'live birth':ab,ti OR 'ongoing pregnancy':ab,ti OR 'clinical pregnancy':ab,ti OR 'spontaneous abortion':ab,ti OR 'recurrent miscarriage':ab,ti OR 'multiple pregnancy':ab,ti OR 'implantation rate':ab,ti OR 'cryopreservation rate':ab,ti OR 'aborted embryo':ab,ti OR 'fetus death':ab,ti OR 'embryo quality':ab,ti OR 'fertilization rate':ab,ti OR 'in vitro fertilization':ab,ti OR 'intracytoplasmic sperm injection':ab,ti) AND 'microrna':ab,ti OR 'untranslated rna':ab,ti OR 'pre mirnas':ab,ti OR 'small rna':ab,ti OR 'small untranslater rna':ab,ti

- 1. Inclusion filters:

('clinical article'/de OR 'comparative study'/de OR 'controlled study'/de OR 'human'/de OR 'major clinical study'/de) AND [embase]/lim NOT ([embase]/lim AND [medline]/lim) AND ('article'/it OR 'article in press'/it)
