# Supplementary material for: The Expression of miRNAs in Human Ovaries, Oocytes, Extracellular Vesicles, and Early Embryos: A Systematic Review
Source: Cells. 2019 Dec 4;8(12):1564. doi: 10.3390/cells8121564 (PMC6952888; doi:10.3390/cells8121564)
Supplement: Supplementary file 1 [file cells-08-01564-s001.zip › Table S1_PICOS_vdef.doc]

**Table S1.** PICOS criteria for inclusion and exclusion of studies.

| **Parameter** | **Inclusion/exclusion criteria** | **Data extraction** |
| --- | --- | --- |
| **Patients** | Well-defined fertile/infertile women or couples attending to IVF/ICSI procedures.  Tissues/cells evaluated: Ovaries and extracellular vesicles, oocytes and embryo | - General information: Location of the study, age, population studied and sample size, and health status. - Fertility parameters: Live birth, ongoing pregnancies, clinical pregnancies, miscarriages, multiple pregnancies, implantation rate, cryopreservation rate, embryo quality and fertilization rate. |
| **Interventions** | No treatments (beyond the intrinsic IVF/ICSI procedures). | Any additional treatment or supplementation reported. |
| **Comparators** | Control group (fertile females or couples). | Numbers of participants in the comparator group. |
| **Outcomes** | Primary outcome:   - miRNA expression level   Secondary outcomes:   - Proportion of aberrant miRNA expression - Gene ontology data (target genes and biological pathways) | Method of identification (microarrays, qRT-PCR, RNAseq, etc.), correction of the results (Bonferroni, FDR, etc.), and normalization method applied. |
| **Study design** | Inclusion: Case-control, cross-sectional, descriptive, observational prospective or retrospective studies.  Exclusion: Clinical trials, animal or in vitro studies, reviews, editorials, opinions and case-reports articles. | Type of study design, subjects in the groups. |
